# Structural basis of Sorcin-mediated calcium-dependent signal transduction

**DOI:** 10.1038/srep16828

**Published:** 2015-11-18

**Authors:** Andrea Ilari, Annarita Fiorillo, Elena Poser, Vasiliki S. Lalioti, Gustav N. Sundell, Ylva Ivarsson, Ilaria Genovese, Gianni Colotti

**Affiliations:** 1 Institute of Molecular Biology and Pathology CNR; Dept. Biochemical Sciences, Sapienza University, P.le A. Moro 5, 00185, Rome, Italy; 2 Centro de Biología Molecular Severo Ochoa, CSIC -Universidad Autónoma de Madrid, Departamento Biología Celular e Inmunología, Cantoblanco; Centro de Investigación Biomédica en Red de Enfermedades Hepáticas y Digestivas (CIBERehd), Madrid, Spain; 3 Department of Chemistry-BMC, Uppsala University, P.O. Box 576, 751 23 Uppsala, Sweden

## Abstract

Sorcin is an essential penta-EF hand calcium binding protein, able to confer the multi-drug resistance phenotype to drug-sensitive cancer cells and to reduce Endoplasmic Reticulum stress and cell death. Sorcin silencing blocks cell cycle progression in mitosis and induces cell death by triggering apoptosis. Sorcin participates in the modulation of calcium homeostasis and in calcium-dependent cell signalling in normal and cancer cells. The molecular basis of Sorcin action is yet unknown. The X-ray structures of Sorcin in the apo (apoSor) and in calcium bound form (CaSor) reveal the structural basis of Sorcin action: calcium binding to the EF1-3 hands promotes a large conformational change, involving a movement of the long D-helix joining the EF1-EF2 sub-domain to EF3 and the opening of EF1. This movement promotes the exposure of a hydrophobic pocket, which can accommodate in CaSor the portion of its N-terminal domain displaying the consensus binding motif identified by phage display experiments. This domain inhibits the interaction of sorcin with PDCD6, a protein that carries the Sorcin consensus motif, co-localizes with Sorcin in the perinuclear region of the cell and in the midbody and is involved in the onset of apoptosis.

Sorcin (Soluble resistance-related calcium binding protein) is a calcium binding oncoprotein expressed at high levels in several human tumours, such as leukaemia, gastric, breast and ovarian cancers^1^,^2^,^3^,^4^,^5^. Sorcin gene is located in chromosome 7, in the same amplicon of other proteins involved in MDR (multidrug resistance) such as ABCB4 and ABCB1 (Mdr1); Sorcin is highly expressed in different chemoresistant cell lines, and its overexpression confers MDR in several tumors^6^,^7^,^8^,^9^,^10^,^11^. Treatment with antisense oligonucleotides increases cell sensitivity for vincristine and other antitumor drugs, suggesting that sorcin might be a useful marker of MDR and a therapeutic target for reversing tumor MDR^12^,^13^.

Sorcin is also considered one of the main regulators of calcium-induced calcium release in the heart^4^,^14^,^15^,^16^,^17^,^18^. Sorcin is one of the most expressed calcium-binding proteins in human cells, especially in the brain and in the heart (PaxDb). In particular Sorcin is one of the most expressed calcium binding proteins in the amygdala, in the prefrontal cortex, in the hypothalamus and in many brain cancers (GeneAtlas U133A, gcrma), such as anaplastic astrocytoma, oligodendroglioma, glioblastoma^19^,^20^,^21^, and is considered a histological marker for malignant glioma^4^.

The Sorcin mechanism of action is not fully understood. However, we have shown that Sorcin is an essential protein, which activates and regulates mitosis and cytokinesis^22^. Our analysis of the interactome of Sorcin revealed calcium-dependent interactions with kinases playing key roles in cell-cycle progression, including Polo-like kinase 1 that phosphorylates Sorcin. In addition, Sorcin silencing blocks cell cycle progression in mitosis and induces cell death. Sorcin localizes at the nucleus, endoplasmic reticulum (ER) and cell membranes during interphase, while during mitosis, Sorcin concentrates in the cleavage furrow (late telophase) and midbody (cytokinesis). Upon calcium binding, Sorcin undergoes large conformational changes, presumably involving exposure of hydrophobic surfaces, that allows it to interact with calcium channels, pumps and exchangers like ryanodine receptors (RyRs), sarco(endo)plasmic reticulum Ca^2+^ ATPase (SERCA), L-type voltage-dependent calcium channels and Na^+^-Ca^2+^ exchangers (NCX), and to regulate them^14^,^15^,^16^,^17^,^23^,^24^. Sorcin regulates calcium homeostasis by binding calcium, binding to and regulating the activity of calcium channels and other proteins; as a consequence, Sorcin increases Ca^2+^ accumulation in the endoplasmic (ER)/sarcoplasmic reticulum (SR) and mitochondria. Sorcin prevents ER stress, and its silencing triggers apoptosis^22^,^25^,^26^. Knockdown of Sorcin in fact results in major defects in mitosis and cytokinesis, increase in the number of rounded polynucleated cells, blockage of cell progression in G2/M, apoptosis and cell death. ER stress is involved in the accumulation and deposits of misfolded proteins in many neurodegenerative diseases; Sorcin interacts in a calcium-dependent fashion with alpha-synuclein and presenilin 2, two proteins involved in the pathogenesis of Parkinson’s disease and Alzheimer’s disease, respectively, *in vitro*, in cultured cells and in human brain^27^,^28^.

From a structural point of view, Sorcin belongs to the small penta-EF-hand (PEF) family^29^. Each monomer of Sorcin homodimer is formed by two domains, i.e. a flexible, glycine-rich N-terminal domain and a C-terminal calcium-binding domain (SCBD), endowed with five EF-hands^30^,^31^. The SCBD can be divided in two regions: EF1–3 (residues 33–134), which binds calcium with high affinity, and EF4–5 (residues 135–198), which mediates dimerization. Upon calcium binding Sorcin undergoes a large conformational change that allows it to bind and regulate a series of target proteins in a calcium-dependent fashion^32^,^33^,^34^. However, the structural basis of its activation and of its ability to establish calcium-dependent interactions with targets is unclear. In addition, the peptide binding motifs are not known, despite the identification of several target proteins.

To investigate the structural basis of Sorcin activation and of selective calcium-dependent signal transduction, we here solved the structure of the apo Sorcin (apoSor) and, for the first time, the structure of Sorcin in the calcium-bound form (CaSor). The structures have been compared with each other and with those of other PEF proteins evaluating: i) the internal variability of Sorcin; ii) the effect of the calcium binding on the single EF-hands; iii) the effect of the calcium binding to the overall protein fold. The comparison reveals potential surfaces of interaction between Sorcin and its targets. We further established consensus Sorcin-binding motifs in absence and presence of Ca^2+^. Finally, we validated interactions between Sorcin and a molecular partner endowed with such sequences, such as PDCD6 (programmed cell death protein 6, formerly called Alg-2), a PEF protein with similar mechanism of activation but different role in the cell with respect to Sorcin, through Surface Plasmon Resonance experiments and in the fibroblasts by co-localization experiments. Our study shed new light on the molecular basis of Sorcin activation, i.e. the structural changes induced by calcium binding in Sorcin, and on Sorcin mechanism of action, i.e. the interaction of Sorcin with its molecular partners, which leads to regulation of cytokinesis, to protection from apoptosis and to the establishment of MDR phenotype.

## Results

### Overall structures and calcium coordination

**Figure 1:**
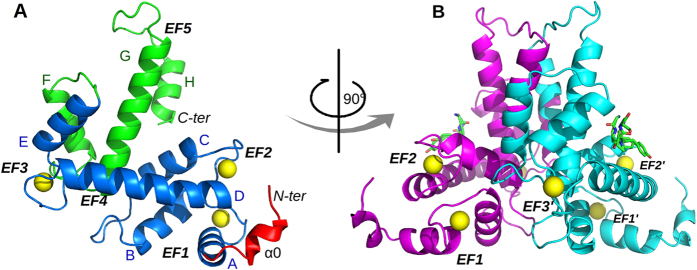
Overall structure of calcium-bound human Sorcin. (**A**) The monomer comprises a part of the flexible N-terminal domain containing an alpha helical region designated α0 (red) and a calcium-binding domain (SCBD) that can be divided in two region: EF1-3 (blue) and EF4-5 (green). Calcium ions (yellow spheres) are bound at EF1, EF2 and EF3. The helices (A-H) and the EF-hands (EF1-5) are indicated. (**B**) Dimerization occurs through the pairing of EF4-5 of two monomers (cyan and magenta). The N-terminal hexapeptide modeled in the structure is shown as green sticks.

We solved structures of Sorcin in complex with calcium (CaSor) and in the apo form (apoSor), at a higher resolution than the one solved by Xie *et al.*^31^. Structures statistics are reported in Table 1. All the structures have the typical fold of the PEF proteins family. Briefly, the monomer is formed by two domains: a Gly-rich N-terminal domain (residues 1–32), partially visible in both apo and calcium-bound structures (residues 30–32 and 26–32, respectively), and a calcium binding domain (SCBD), containing eight α-helices (A-H) organised in five calcium binding motifs (EF1-EF5). Two helices are very long and connect two adjacent EF hands: the D-helix (hD) is common to EF2 and EF3, while the G-helix is common to EF4 and EF5 (Fig. 1). EF1 is structurally coupled with EF2, and EF3 is paired with EF4. Sorcin dimerization occurs by pairing of the EF5 of two monomers.

**Figure 2:**
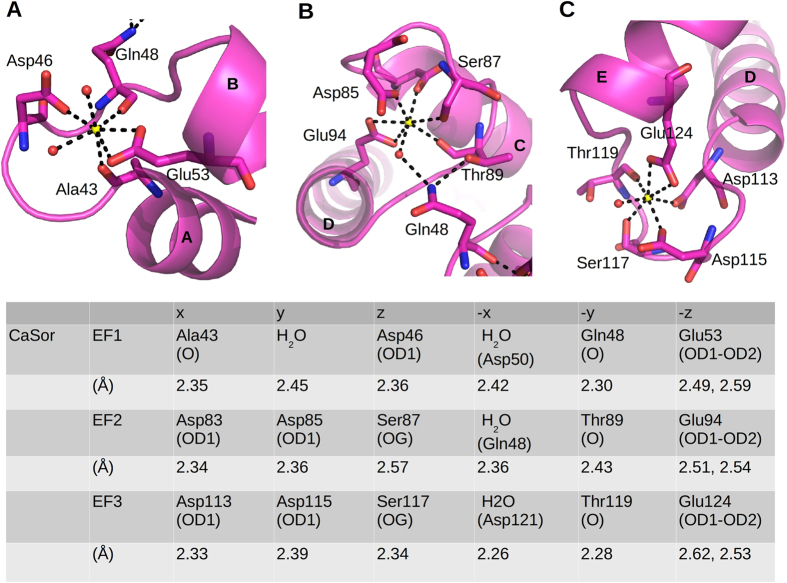
Calcium coordination in Sorcin. Close-up of Ca^2+^ binding sites in EF1 (**A**), EF2 (**B**) and EF3 (**C**) reveals the classical pentagonal bipyramidal geometry. The involved residues are shown as sticks, water molecules as red spheres and calcium ions as yellow spheres. Ligand positions and coordination distances are listed.

In CaSor, Ca^2+^ is bound at EF1, EF2 and EF3 and it is hepta-coordinated in a classical pentagonal bipyramidal configuration (Fig. 2 and Table S1). EF1 and EF2 are coupled by Gln48, which coordinates the EF1-bound Ca^2+^, whereas in EF2 is hydrogen-bound to Thr89.

### Comparison of human Sorcin structures: conformational changes induced by calcium binding

**Figure 3:**
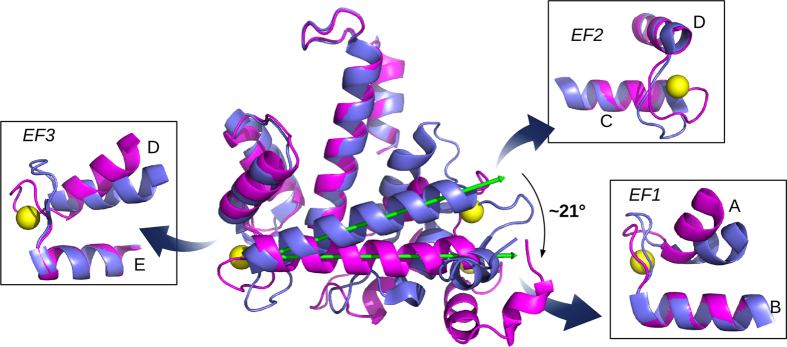
Conformational changes induced by ion binding. The superposition of CaSor (magenta) and apoSor (blue) reveals the conformational variation induced by calcium (yellow spheres). The green arrows represent the axis of the D helix in the two structures: the binding of three Ca^2+^ to each Sorcin monomer causes a large movement of the D helix that drags the EF1-EF2 region. The panels illustrate the changes of EF1, EF2 and EF3 taken alone, analysed aligning the C-terminal helix for each EF-hand: EF1 and EF3 open upon Ca^2+^-binding, while EF2 is almost unchanged.

The comparison between all the known human Sorcin structures (apo human Sorcin, PDB code: 1JUO; apo-F112L human Sorcin mutant, PDB code: 2JC2; apoSor, PDB code: 4UPG; CaSor, PDB code: 4USL) shows that the EF1–3 region is more flexible than the EF4-EF5 region and that a large conformational change in the EF1-EF2 subdomain and EF3 is visible upon calcium binding to the first three EF hand motifs^16^,^31^. Indeed, the rmsd values (Table S2) measured by superimposing CaSor to the apoSor clearly indicate that upon calcium binding Sorcin undergoes a large conformational change, mainly involving EF1, EF2 and EF3 (Table S2, Fig. 3). As shown in Fig. 3, calcium binding to EF1, EF2 and EF3, i.e. the three EF hands with the highest affinity for the cation^33^, induces a large displacement (of about 21°) of the D-helix). The comparison between apoSor and the calcium-bound Sorcin structures sheds light on the mechanism of cation-mediated structural changes of Sorcin, which is fundamental for the comprehension of its function. The binding of calcium at the EF3 loop causes the movement of the three ligands Asp113, Asp115 and Ser117 towards the bidentate Glu124 ligand in the E-helix. Thus, the loop undergoes a rearrangement and may act as a lever dragging the long and rigid D-helix away from the E-helix. As a result, EF3 acts as a pivot: the first half of the calcium binding domain (formed by A-, B-, C- and D-helices) rotates and moves away from the second half (formed by the E-, F-, G- and H-helices), which is the dimerization subdomain and forms the stable Sorcin dimeric interface.

### Calcium binding and mechanism of activation: comparison with PDCD6 (Alg-2)

Sorcin binds calcium at EF1, EF2 and EF3 hands. In order to investigate the role of each calcium binding site, we analysed each EF hand separately and evaluated the structural variation induced upon ion binding. For the sake of simplicity, the local conformational change has been evaluated by measuring the variation of the angle between the two helices of each EF-hand motif in the structures of Sorcin and of other PEF proteins, whereas the overall conformational change has been evaluated by measuring the variation of the angle between the D- and G-helices, which takes into account the movement of the EF1-EF2 sub-domain and EF3 with respect to the EF4 and EF5 hands (Table 2).

The PEF proteins are divided in two sub-groups on the basis of the residues present on the EF1 loop; in the group I the EF1 loop is formed by 11 residues whereas in the group II, to which Sorcin belongs, the EF1 loop is formed by 12 residues^35^. The proteins for which both the structures in the apo form and in the presence of a saturating amount of calcium were determined and deposited in the PDB, have been chosen to be compared with Sorcin structures: human PDCD6 (PDB codes: 2ZND, 2ZN9^36^), representative of the group I, and rat m-calpain PEF domain dVI (PDB codes: 1AJ5, 1DVI^37^), representative of the group II.

This analysis shows that in Sorcin both EF1 and EF3 hands are widely opened upon calcium binding (variation of the angle between the helices of the EF hand, *θ* = +18.4° and +14.5° respectively), while EF2 displays only a minimal variation (*θ* = −3°).

The comparison with other PEFs is informative and interesting. PDCD6 binds Ca^2+^ (or Zn^2+^) at EF1, EF3 and EF5 but the local conformational changes (*θ* = +3.2°, +4.8°, −8.6°) are smaller than those observed in Sorcin. On the contrary calpain-dVI, which binds Ca^2+^ at the same sites as Sorcin (plus a fourth Ca^2+^ not bound at an EF-hand), shows a similar change for EF1 and EF3 (*θ* = ~20°, ~14°) and an evident opening of EF2 (*θ* = ~10°). The open question is how the local conformational changes described so far can be transmitted to the overall structure.

As already discussed, in Sorcin the opening of EF3 causes the exposure of a hydrophobic surface with the shift of the EF1-EF2 sub-domain and EF3 with respect to the EF4-EF5 sub-domain (Fig. 3). The variation of the angle between D-helix and G-helix can be used as an indicator of such movement and, as already stated, is of about 21°. As shown by the structural analysis of CaSor the opening of EF1 causes the exposure of an additional hydrophobic surface. The opening of EF1 and EF3 causes the exposure of two distinct hydrophobic surfaces that likely mediate the interaction of Sorcin with its molecular partners.

Calcium binding to PDCD6 does not cause large conformational changes. Indeed, the superimposition between the calcium bound and the apo PDCD6 monomers using the Cα atoms, yields a rmsd = 1.20 Å and the angle between the D- and G-helices varies only by 2°; the main effect is observed on the EF5 hand where the binding of calcium causes a slight twisting movement and opening of the dimer. Interestingly the hD-hG angle in both the apo and the calcium bound form of PDCD6 has a value similar to that measured in CaSor (75°–80° vs 78°) suggesting that the two proteins may share similar target binding sites, with the difference that PDCD6 sub-domains are always in the active overall conformation and therefore only minor variations are necessary to allow target binding. Ca^2+^-dependent activation of PDCD6 is ascribed to the movement of the side chain of Arg125, belonging to the loop between EF3 and EF4, that uncovers and makes accessible the hydrophobic pocket already present in the apo form^36^.

### Analysis of Sorcin solvent-accessible surface areas

**Figure 4:**
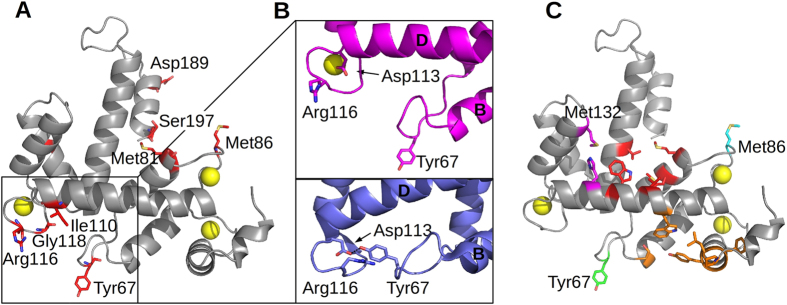
Solvent accessible surface analysis and hot-spots prediction. (**A**) The residues that upon calcium binding become more accessible (SASA increase higher than 30%) are mapped as red sticks on CaSor structure; Tyr67 and Met86 show the strongest variation. (**B**) In apoSor (blue) Tyr67 forms a hydrogen bond with Asp113. In CaSor (magenta) the hydrogen bond is broken and the loop moves away together with helix B and the EF1-EF2 region. (**C**) Hotpatch analysis identified 3 pockets (pocket 1, magenta; pocket 2, red; pocket 3, orange) likely mediating protein-protein interactions.

The analysis of solvent accessible surface areas has been performed with areaimol (CCP4 suite, http://www.ccp4.ac.uk/html/areaimol.html) and shows that upon calcium binding there is an increase in the exposed surface areas of several residues. The residues with a difference in SASA (Solvent Accessible Surface Areas) higher than 30% between the apo and the calcium bound form of Sorcin are Tyr67, Ser80, Met81, Met86, Ile110, Arg116, Gly118, Ser143 and Ser197 (Table S3, Fig. S1). As shown in Fig. 4A, the residues displaying the highest SASA (higher than 30%) are located in the loop preceding the C helix (hC), in the EF2 loop (which follows the hC), in the C-terminal part of the D-helix and in the EF3 loop; all these structural features present a wide calcium-dependent rearrangement.

Even if, as shown in Table 2, ion binding has almost no effect on the relative position of helices C and D of EF2 hand, upon ion binding there is a reorganization of the last part of the helix C containing Met81 and of the loop 83–91 containing Met86, which become exposed to the solvent. Tyr67 is placed on the loop between helices B and C, and in apoSor it is hydrogen bonded to Asp113 of the EF3 loop and is partially covered by it. Upon calcium binding this interaction is broken since Asp113 participates in ion coordination; the rearrangement of the EF3 loop causes also the exposure of Arg116 (Fig. 4, panel B).

We further analysed the CaSor structure using the Hotpatch server (http://hotpatch.mbi.ucla.edu/) in order to identify unusual hydrophobic patches likely mediating protein-protein interactions between Sorcin and its molecular partners^38^. The Hotpatch analysis highlights that besides Met86 (cyan) and Tyr67 (green), each Sorcin monomer has two significant regions consisting of three different zones, shown in Fig. 4C. The pink one includes His108 and Met132 (pocket 1), the red one Met81, Val101, Trp105, Val164 (pocket 2) and the orange one Ala26, Phe27, Pro28, Pro34, Leu35, Tyr36, Gly37, Tyr38, Ser61, Trp99 (pocket 3). Interestingly, these clusters are found in the areas most affected by calcium dependent structural changes, namely EF1 (orange residues) and EF3 (red and pink residues). Moreover, both areas include tryptophan residues (Trp99 and Trp105) strongly conserved among the PEF protein family members. Supporting the importance of these regions in ligands binding, Colotti and coworkers previously demonstrated that mutation of Trp105 impairs the capacity of Sorcin to recognize and interact with RyR2 and annexin 7 at physiological calcium concentrations^39^.

### Analysis of N-terminal peptide-Sorcin interaction and comparison with the PDCD6-Alix structure

**Figure 5:**
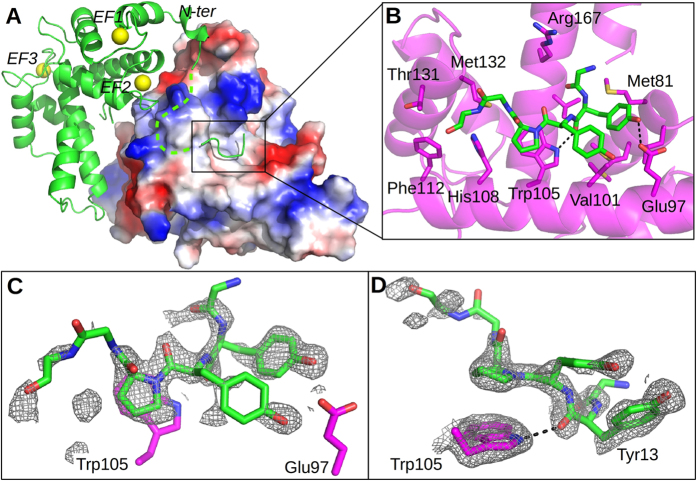
Interaction between Sorcin and the N-terminal peptide. (**A**) The electrostatic surface potential (blue-positive, red-negative) of CaSor dimer is shown. The hydrophobic surface corresponding to pockets 1–2 accommodates the 12-GYYPGG-17 peptide (green) plausibly belonging to an adjacent Sorcin molecule in the crystal (green cartoon); the residues 11–25 are not visible (green dashes). (**B**) Close-up of the peptide-binding region: the peptide is shown as green sticks, the residues interacting with the peptide are depicted as magenta sticks, and the hydrogen bonds between Trp105-Tyr17 and Glu97-Tyr17 are indicated as black dashes. (**C**,**D**) Two views of the electron density map of the peptide (2Fo-Fc, contoured at 1σ).

The analysis of the CaSor structure reveals the presence of an electron density peak in the cavity formed upon calcium binding and the consequent tilt of the D-helix. We fitted this electronic density map with the GYYPGG hexapeptide belonging to the N-terminal region of Sorcin (residues 12–17). The same region was thought to interact with PDCD6 N-terminal peptide by Jia *et al.*^40^; Suzuki *et al.* demonstrated that it probably was PEG^36^. We can exclude PEG binding to Sorcin structure: the Fo-Fc and 2Fo-Fc electron density maps shows clearly the presence of a short peptide containing side chains with a very well resolved proline residue clearly visible in the structure (12-GYYPGG-17; Fig. 5, Fig. S2), belonging to a different dimer. The interacting surface between the N-terminal peptide and Sorcin was analysed using the program ePISA (http://www.ebi.ac.uk/msd-srv/prot_int/cgi-bin/piserver). The residues buried at the interface between peptide and Sorcin are: Met78, Met81, Leu82, Glu97, Ala100, Val101, Gly104, Trp105, His108 placed on the D helix; Phe112 on the EF3 loop; Thr131, Met132, on the EF4 loop; Val164, Arg167, and Asp171 on the G helix. Trp105, Glu97 and Arg167 form hydrogen bonds with Tyr13 and Tyr14 of the peptide (OH Tyr13-OE2 Glu97 = 2.78 Å; O Tyr13-NE1 Trp35 = 2.74 Å; O Tyr14-OE2 Glu97 = 2.90 Å) (Fig. 5B). The residues laying on the D helix play a major role in interacting with the N-terminal peptide; in particular, Trp105 establishes a strong stacking interaction with Pro15 and is hydrogen bonded to the carbonyl group of Tyr13, determining the orientation of the peptide into the pocket which is opposite to that of Alix in PDCD6 (see below). These residues belong to pockets 1 and 2, identified by Hotpatch analysis (Fig. 4C).

The calcium-dependent movements of the EF3 loop determine conformational changes of different extent in the PEF proteins (Fig. S3). As shown in Table 2, the opening of D and G helices opening is quite similar in zinc-bound PDCD6 complexed with Alix and CaSor complexed with the N-terminal peptide (the measured angles between the D and G helices are 78° and 82° respectively). As described by Suzuki and co-workers^36^, the peptide binds to two juxtaposed hydrophobic pockets (1 and 2), which hold PPYP and YP, respectively. The residues lining the pocket 1 are Gly123, Tyr124, Arg125, Thr162, Phe165, Gln172, Gly174 and the residues lining the pocket 2 are Met71, Phe72, Tyr91, Asp94, Trp95, Phe122, Gln159. More recently a third pocket has been identified in PDCD6 (Pocket 3) capable to bind the type 2 motif PXPGF present in Sec31A^41^. Pocket 3 is formed by residues belonging to EF1, the EF1–EF2-connecting loop, EF2, EF3 and EF4 (Phe27, Val31, Val35, Leu48, Ala51, Leu52, Ser53, Gly55, Trp57, Phe85, Val88, Trp89, Ile92, Thr93, Gln96, Phe99, Gly108, Met109, Phe148). Our structures obviously do not contain peptides bound to this pocket, but we cannot rule out the possibility of peptide binding to this site.

**Figure 6:**
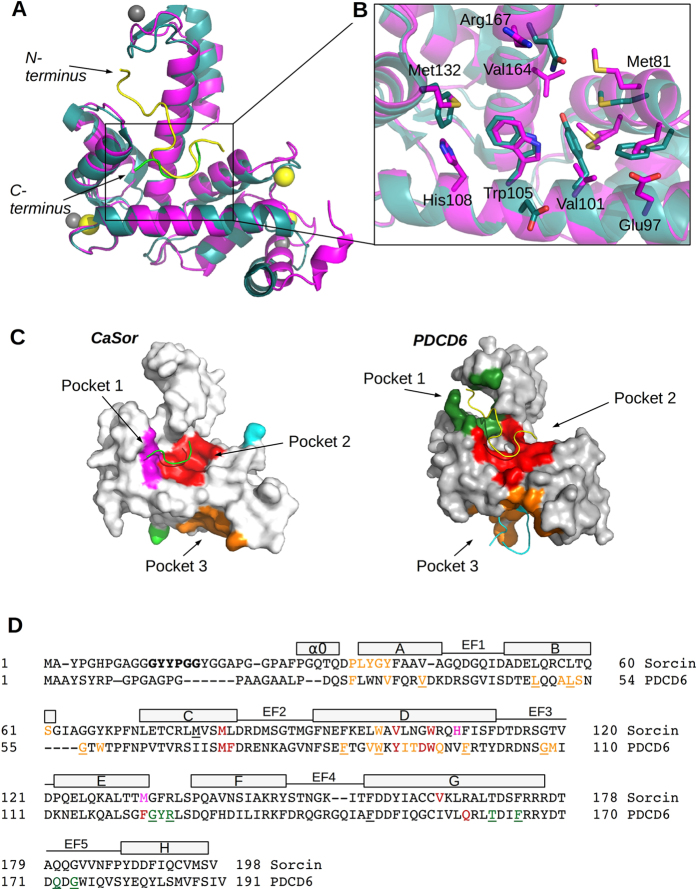
Peptide binding and pockets comparison in CaSor and PDCD6. (**A**) The superimposition of CaSor (magenta; calcium in yellow, peptide in green) and PDCD6 (teal; zinc in grey) in complex with Alix peptide (yellow) shows that the protein have a similar conformation and that both peptides bind in pocket 2 but in opposite direction, as indicated by arrows. (**B**) The main residues lining pocket 2 are shown (Sorcin numbering); note the presence of Val101 instead of Tyr91. (**C**) The pockets predicted by Hotpatch in CaSor (left, same color code as Fig. 4C) and the pockets found in PDCD6 (right) by co-crystallization with Alix peptide (yellow) and Sec31A peptide (cyan) are mapped on the surfaces and indicated by arrows. (**D**) Structural alignment of Sorcin and PDCD6. The residues corresponding to the hexapeptide are in bold. The residues lining the pockets are colored accordingly and the ones present in both sequences are underlined.

The superimposition between Alix-bound PDCD6 and CaSor clearly shows that these pockets are present both in both proteins, and the structural alignment reveals that many of the residues lining the pockets are conserved in Sorcin (underlined residues in Fig. 6D). In particular, relevant conserved residues are: Trp95 (Trp105, Sorcin numbering) which establishes a strong stacking interaction with the proline of the N-terminal peptide and in Sorcin was demonstrated to be necessary for the interaction with its molecular partners; Arg125 (Arg135, Sorcin numbering) whose mutation to alanine caused a loss of binding ability of PDCD6 to Alix; and Met71 (Met81, Sorcin numbering) which was demonstrated to be one of the residues changing more its SASA and that was suggested by Hotpatch analysis to be one of the residues mediating Sorcin interactions. A significant difference concerns Tyr91 that in Sorcin is substituted by Val101, allowing the interaction already described between Trp105 and Tyr13 (Figs 5B–D and 6B) that partially explains the opposite orientation of the N-terminal peptide in Sorcin with respect to Alix peptide in PDCD6.

Todd and coworkers showed that calpastatin interacts with residues belonging to A and C helices of the calpain-dVI in the open conformation^42^, corresponding to pocket 3 identified by Hotpatch analysis in Sorcin (data not shown). In particular two hydrophobic residues of calpastatin, namely Leu606 and Phe610, were found to be necessary for the interaction with calpain. The pocket where Leu606 binds is lined by several bulky aromatic residues (Phe99, Phe162 and Trp166) and a variety of other hydrophobic residues (Leu102, Leu106, Ile121 and Val125); Phe610 binds to a large hydrophobic pocket formed by the B helix and N-terminal part of the D helix and is in close van der Waals contact with residues His129 and Gln173. Two of the three aromatic residues binding calpastatin belonging to the D helix are conserved in Sorcin (Phe95, corresponding to Phe162 in calpain-dVI, and Trp105 corresponding to Trp166 in calpain-dVI), whereas the residues indicated to line the hydrophobic pocket where Leu606 of calpastatin binds are not conserved but anyway are substituted by hydrophobic residues (Leu106 is substituted by an alanine residue, Ile121 is substituted by a leucine and Val125 is substituted by a leucine). The residues His129 and Gln173 are not conserved in Sorcin whereas also in Sorcin the B helix and N-terminal part of the D helix are lined by residues forming a hydrophobic pocket, namely Phe156 (Phe224 in calpain-dVI) and Leu102 (substituted by Ile169 in calpain-dVI).

### Phage display selection in presence of EDTA and Ca^2+^

**Figure 7:**
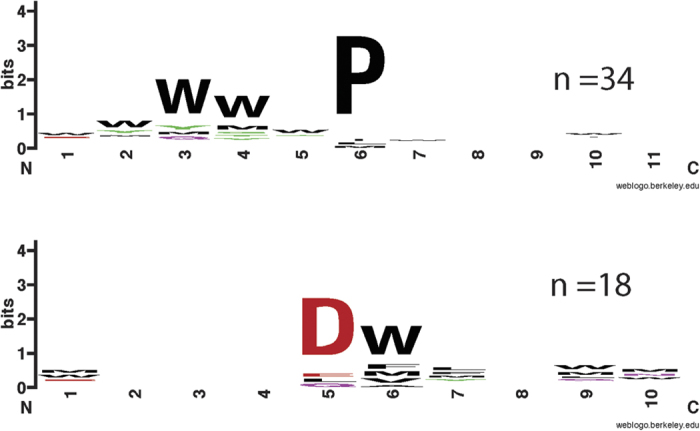
WebLogo outputs of consensus peptide motifs identified through peptide phage display. (**A**) The Φ/Gly/Met-Φ/Gly/Met-x-P motif is based on 34 unique peptide sequences, of which 20 were obtained from a phage selection performed in the presence of 1 mM Ca^2+^. (**B**) The acidic-Φ motif is from 18 unique peptides of which 16 were selected in presence of Ca^2+^.

To investigate if the structural changes conferred by the Ca^2+^ binding translate into specificity changes, we used Sorcin as a bait protein against a highly diverse M13 phage display library that displays 16mer peptides on the major coat protein p8. Selections were performed in the presence of EDTA (1 mM) or Ca^2+^ (1 mM) and were in both cases successful as judged by pooled phage ELISAs (i.e. signal to background >2). Sequencing of individual clones (38 and 20 clones from the selections in presence or Ca^2+^ and EDTA, respectively) revealed that the majority of ligands contains a conserved Pro and that the main consensus motif under both conditions is a relaxed Φ/Gly/Met-Φ/Gly/Met-x-P, where Φ/Gly/Met is an aromatic residue (Trp, Tyr or Phe) or a Gly or Met residue, and x is any amino acid (Fig. 7A, Fig. S4). The consensus sequence agrees with the GYYPG peptide belonging to the Sorcin N-terminal domain, identified in the Sorcin binding site in our crystal structure. In addition, there is a set of peptides that lack a clear Φ/Gly/Met-Φ/Gly/Met-x-P motif but instead hold an acidic-Φ motif (Fig. 7). Such peptides are more frequently observed in presence of Ca^2+^ (47% of sequenced peptides) than in the presence of EDTA (15% of sequenced peptides). In a cellular context, likely Sorcin can establish interactions with a variety of ligands containing the main Φ/Gly/Met-Φ/Gly/Met-x-P motif, or the acidic-Φ motif found in intrinsically disordered regions of target proteins. Such interactions might be facilitated by the exposure of hydrophobic binding surface in Sorcin upon Ca^2+^ binding, as suggested by the structure. However, peptide binding might occur also in absence of Ca^2+^ if the preferred target is readily available as in the high avidity p8 phage display. Indeed, the presence of a high affinity ligand might shift the equilibrium towards the open conformation. Further detailed mechanistic studies should shed light on this issue.

### Selective calcium-dependent interactions between Sorcin and targets

The interactions between Sorcin and PDCD6 (programmed cell death protein 6) (formerly called Alg-2) a member of the PEF protein family, endowed with Sorcin N-terminal consensus binding motifs was tested by both SPR and colocalization experiments. PDCD6 has a role in the mechanisms of apoptosis onset, and was shown to interact with N-terminal peptide of annexin 11 in the presence of 50 μM Ca^2+^, with a higher affinity than Sorcin^43^. PDCD6 has 36% identity with respect to Sorcin, displays similar structure and displays residues as Trp95 Arg125 and Met71, conserved also in Sorcin, which allow its interaction with Alix^36^ and potentially with the sorcin N-terminal domain. Moreover, PDCD6 displays an N-terminal domain similar to that of sorcin, and containing Φ/Gly/Met-Φ/Gly/Met-x-P sequences identified as Sorcin-interacting motifs.

**Figure 8:**
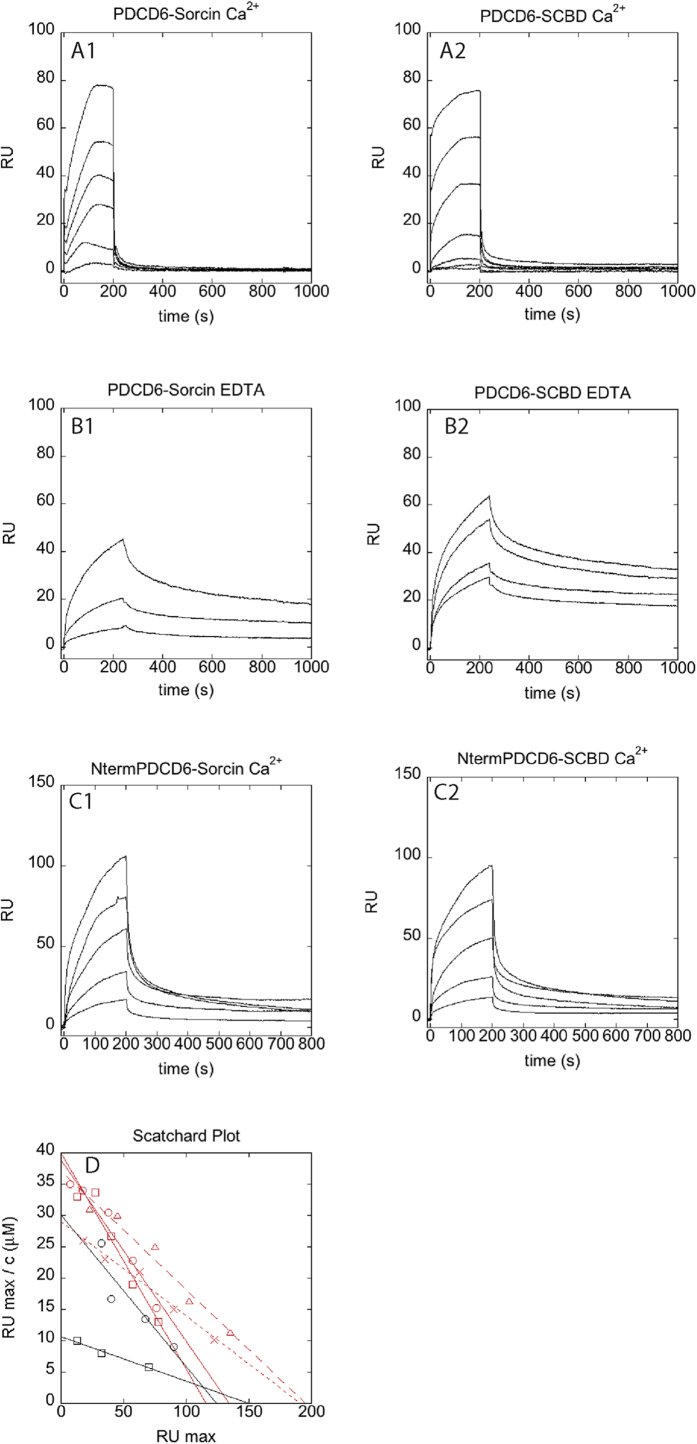
Interaction of Sorcin with full-length PDCD6 and N-terminus of PDCD6. (**A**) Sensorgrams showing the interaction between PDCD6, immobilized on a COOH5 chip and different concentrations of Sorcin (left panel; from bottom to top: 200 nM, 400 nM, 800 nM, 1.5 μM, 3 μM, 6 μM), and SCBD (right panel; from bottom to top: 50 nM, 100 nM, 200 nM, 500 nM, 1 μM, 2.5 μM, 5 μM), in the presence of 100 μM calcium. (**B**) Sensorgrams showing the interaction between PDCD6, immobilized on a COOH5 chip and different concentrations of Sorcin (left panel: from bottom to top: 1.3 μM, 4 μM, 12 μM), and SCBD (right panel: from bottom to top: 1.25 μM, 2.5 μM, 5 μM, 10 μM), in the presence of 1 mM EDTA. (**C**) Sensorgrams showing the interaction between the N-terminal domain of PDCD6, immobilized on a COOH5 chip and different concentrations of Sorcin (left panel; from bottom to top: 750 nM, 1.5 μM, 3 μM, 6 μM, 12 μM), and SCBD (right panel; from bottom to top: 750 nM, 1.5 μ μM, 3 μM, 6 μM, 12 μM), in the presence of 100 μM calcium. (**D**) Scatchard plots of the experiments in Fig. 8A–C, and linear fittings. Red squares: PDCD6-Sorcin interaction in the presence of 100 μM calcium; red circles: PDCD6-SCBD interaction in the presence of 100 μM calcium black squares: PDCD6-Sorcin interaction in the presence of 1 mM EDTA; black circles: PDCD6-SCBD interaction in the presence of 1 mM EDTA; red triangles: N-terminal domain of PDCD6-Sorcin interaction in the presence of 100 μM calcium; red crosses: N-terminal domain of PDCD6-SCBD interaction in the presence of 100 μM calcium.

**Figure 9:**
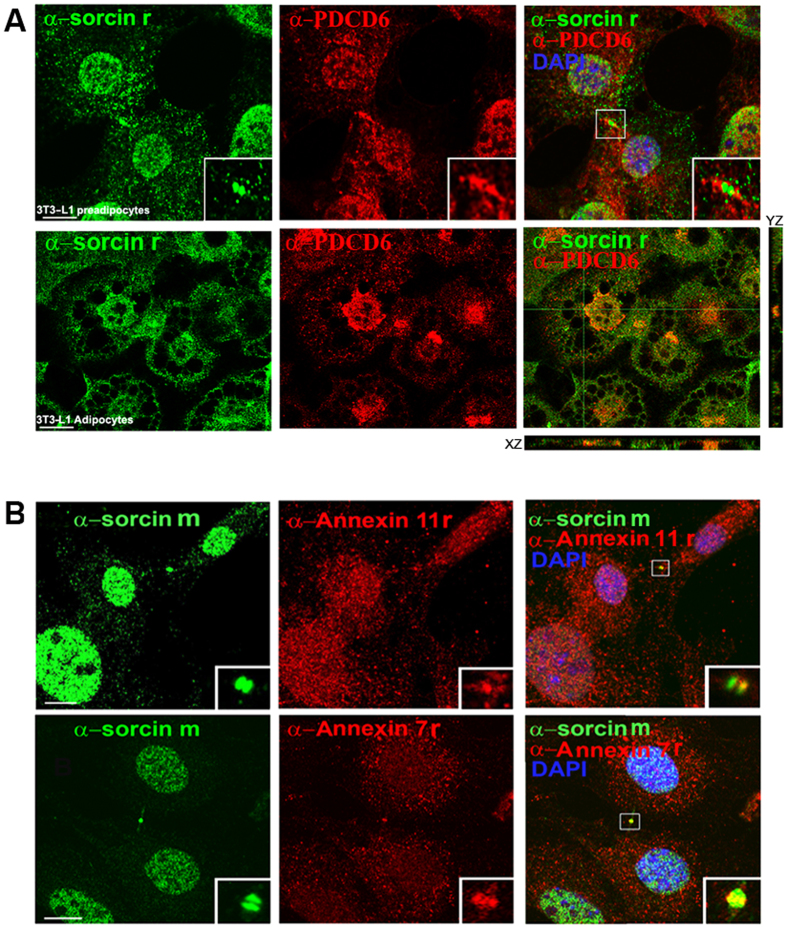
Colocalization of Sorcin with PDCD6 and annexins 7 and 11. (**A**) Experiments showing co-localization between sorcin (rabbit α-sorcin, green) and PDCD6 (mouse α-PDCD6, red), in 3T3-L1 preadipocytes in cytokinesis (top panel) and differentiated 3T3-L1 adipocytes (bottom panel) in X and Z axes. Bars: 10 μm. Note the colocalization in the midbody of 3T3-L1 preadipocytes and in the perinuclear region of adipocytes. (**B**) Experiments showing co-localization between Sorcin (mouse α-sorcin, green) and annexin11 (top panel: rabbit α-annexin11, red), or annexin7 (bottom panel: rabbit α-annexin7, red), in 3T3-L1 preadipocytes in cytokinesis. Bars: 10 μm. Note the colocalization in the midbody (arrows and insets).

SPR experiments show that both the whole Sorcin and SCBD are able to interact with PDCD6 in the presence of calcium, with a K_D_ = 3.5 μM (Fig. 8A). In the presence of EDTA, SCBD interacts with PDCD6 with a K_D_ = 5 μM whereas the calcium-free Sorcin interacts with PDCD6 with an even lower affinity (K_D_ = 12 μM Fig. 8B). Both association and dissociation are faster in the presence of calcium than in the presence of EDTA. The N-terminus has therefore an inhibitory activity in Sorcin-PDCD6 interaction at low calcium concentrations. Additionally, both Sorcin and SCBD interact with the N-terminal domain of PDCD6, with K_D_ = 5 μM for Sorcin and K_D_ = 6 μM for SCBD (Fig. 8C). Partial colocalization between Sorcin and PDCD6 takes place in perinuclear regions of differentiated 3T3-L1 adipocytes and in the midbody of 3T3-L1 preadipocytes (Fig. 9A). Sorcin also colocalizes with annexin 7 and annexin 11, which possess N-terminal domains containing Φ/Gly/Met-Φ/Gly/Met-x-P sequences, in the midbody of 3T3-L1 preadipocytes (Fig. 9B).

## Discussion

Sorcin is overexpressed in several tumor cells as an adaptive mechanism to prevent ER stress and escape apoptosis triggered by chemotherapeutic agents, prompting its further investigation as a novel molecular target to overcome MDR^26^. The present study discloses the structural changes induced by calcium binding in Sorcin and sheds light on the mechanism of interaction of Sorcin with its molecular partners, and thereby on Sorcin-dependent regulation of cytokinesis and establishment of MDR phenotype.

The binding of calcium to Sorcin promotes a large conformational change, which involves a tilt of the D-helix with respect to the G-helix, with EF3 acting as a hinge (Fig. 3). This movement, as displayed in Table 2, is about 21° and is the highest among the PEF family members. Ca^2+^ binds to the three high affinity calcium binding sites EF1, EF2 and EF3. Calcium binding to EF1 and EF3 causes a large reorganization of the EF hands and the consequent movement of the EF helices one in respect to the other, whereas calcium to EF2 determines only a local reorganization of the residues of the EF2 loop.

The large D-helix displacement causes the rotation of the EF1-EF2 subdomain (containing A, B, C and D helices) with respect to the EF3-EF4-EF5 subdomain (containing E, F, G and H helices). From the structure it is not possible to understand which is the calcium binding EF-hand endowed with the highest affinity. However, calcium binding studies performed in solution by spectroscopic methods on wt Sorcin and site specific mutants clearly showed that EF3 is the highest affinity site, because when Glu124 (the bidentate ligand in EF3) is mutated the affinity for calcium of the entire protein dramatically decreases^33^. Mutation in the bidentate Glu94 or Glu53 (placed on the EF2 and EF1 loops) has milder effects on overall Sorcin calcium affinity and on Sorcin ability of interacting with target proteins. The superimposition (Fig. 3) between the CaSor and apoSor shed light on the mechanism of Sorcin activation induced by the calcium binding to EF3: the binding of the cation promotes the movement of three calcium ligands (Asp113, Asp115 and Ser117) towards the E-helix where Glu124, the bidentate ligand (Fig. 3), is located and consequently, a large movement of the D helix with respect to the E helix (about 15°, see Table 2). This movement is transmitted to the EF1-EF2 subdomain via the Tyr 67 placed in a strategic position, in the middle of the loop connecting EF1 to EF2, and anchoring the EF1-EF2 subdomain to the EF3 loop. The binding of calcium to EF3 causes the breakage of the hydrogen bond between Asp113 (one of the calcium ligands of EF3) and Tyr67 and consequently the hC and hB helices are free to move.

The structural analysis shows that upon calcium binding there is the formation of two possible interaction sites per monomer. A site (pocket 1 + 2 in the Hotpatch analysis) is lined by residues of the C-terminal part of the D helix and residues of the EF3-hand, which in PDCD6 is the site of interaction with Alix. Another potential site (pocket 3 in the Hotpatch analysis) involves the EF1 and EF2, which in calpain-dVI bind calpastatin and in PDCD6 binds Sec31A^41^,^42^. Interestingly, the interaction with a N-terminal peptide is similar to that of the peptide bound by PDCD6, and the stacking interaction with the proline of the peptide and Trp105 is conserved between the two structures. It confirms that Trp105 is a residue with a key role in the recognition of hydrophobic Pro-containing peptides in both PEF proteins. The peptide phage display experiments confirm that Sorcin binds preferentially Pro-containing peptides. This analysis further suggests an alternative binding motif (acidic-Φ). The interaction with the latter motif appears to be promoted by calcium binding. Positively charged residues located in the EF-loop (Arg135) and at the G-helix (Arg174, Arg175, Arg176), close to pocket 2, or His108 in pocket 1 are possibly responsible for the binding of these peptides.

Thus, Sorcin may interact via both its N-terminal domain and its SCBD domain with proteins containing the Φ/Gly/Met-Φ/Gly/Met-x-P consensus motif, such as TRAP1, a global regulator of tumor metabolic reprogramming^44^, which contains a SIFYVPDMKP sequence that includes the consensus motif. In this framework, the SPR experiments carried out to study the interaction of Sorcin and SCBD with PDCD6 demonstrates that the interaction takes place via sorcin C-terminal domain, because SCBD retains the ability to interact with the target. Moreover, the SPR experiments show that the affinity between SCBD and PDCD6 is higher than between PDCD6 and Sorcin, demonstrating that the N-terminal domain partially inhibits this interaction at low, physiological calcium concentration (in the presence of EDTA). These data shed light on the possible role of different splicing version of Sorcin, which mostly differ for the length of their N-terminus, and can interact in different fashion with different targets: the short, so called mitochondrial 19-kDa Sorcin B-isoform lacks the residues 2–17 (AYPGHPGAGGGYYPGG), which include the region that, in the crystal structure, interacts with the hydrophobic calcium-dependent pocket 1. The Sorcin B-isoform may therefore able to interact with targets with higher affinity than the A-isoform. The interaction of the N-terminal domain with the C-terminal domain of a neighbouring dimer may also be responsible for Sorcin oligomerization (Fig. 6).

Partial colocalization between Sorcin and PDCD6 takes place in perinuclear regions of differentiated 3T3-L1 adipocytes and in the midbody of 3T3-L1 preadipocytes (Fig. 9). This interaction may be important for the formation of this transient structure in the latest stage of cytokinesis. A competition between Sorcin and PDCD6 for similar targets may also take place, via pockets exposed to solvent upon calcium binding. Interestingly PDCD6 has a mechanism of activation based on the switch of an arginine residue and this residue is conserved also in sorcin (Arg135, Sorcin numbering)^36^. Calcium-dependent interactions with annexin 7 and annexin 11 in the midbody may also take place with the same mechanism.

In conclusion, in this paper the Ca^2+^-induced conformational change in sorcin has been investigated for the first time. We demonstrate that this change involves a large movement of the D-helix, which takes place in this extent only in sorcin among PEF proteins. Moreover, the study reported here gives the unique opportunity to visualize the interaction between the two sorcin domains: SCBD and the N-terminal domain. Finally, we demonstrate that the interaction between sorcin and its molecular partners may take place via both the SCBD and N-terminal domain and that this latter domain may exert a regulative role by inhibiting in some extent the binding of sorcin to its protein targets.

## Methods

### Protein crystallization, data collection and data reduction

Recombinant proteins (human Sorcin, SCBD and PDCD6) were expressed in pET vectors (Novagen) in *E. coli* BL21(DE3) cells, purified according to published procedures^18^ and dialysed in 20 mM Tris-HCl, at pH 7.5. Automated crystallization screening and by-hand optimization were carried out at 298 K by the hanging-drop vapor diffusion method. Since sorcin precipitates when it is saturated with calcium, we performed the starting crystallization trials with commercial screens adding 5 mM CaCl_2_ in the reservoir before mixing the crystallization drops. ApoSor resulted to be rather prone to crystallization even in the presence of calcium; therefore, in order to discriminate between apoSor and CaSor crystals we performed all the crystallization trials in double, with and without calcium. The apoSor crystallization trials were performed using a protein sample concentrated to about 10 mg/ml. Aliquots (1 μl) of the protein sample were mixed with an equal amount of reservoir solution containing 20–22% (w/v) polyethylene glycol 4000, 0.3–0.5 M ammonium sulfate. Crystals grew in 2 weeks and reached dimensions of 0.1 mm × 0.2 mm × 0.3 mm.

Crystals of CaSor were obtained by mixing 1 μl of protein solution, concentrated to about 15 mg/ml, using a reservoir solution containing: 20–25% (w/v) polyethylene glycol 3350, 0.5 M lithium sulfate, 0.1 M Tris-HCl at pH = 8.5 and 5 mM CaCl_2_. For data collection, apoSor and CaSor were cryo-protected in a solution containing 80% (v/v) of mother liquor and 20% (v/v) polyethylene glycol 200. The crystals were mounted in nylon loops and flash frozen by quick submersion into liquid nitrogen and transported to the synchrotron-radiation source. Single-wavelength data sets (λ = 1 Å) were collected from crystals of apoSor and CaSor at the 5.2 R beamline of the Synchrotron Radiation Source ELETTRA (Trieste, Italy), using a Pilatus 2 M detector at a temperature of 100 K. The data sets were processed with XDS^45^ and scaled with XSCALE^45^. Crystal parameters and data collection statistics for the measured crystals are listed in Table 1.

### Structure solution and refinement

The structure of apoSor was determined by molecular replacement with the program MOLREP^46^ (CCP4 suite) using the structure of the calcium-free human Sorcin (PDB entry 1JUO)^31^ as search model.

The case of CaSor was more complex: first we solved the structure of SCBD (Sorcin Calcium Binding Domain) with calcium (Ca-SCBD, data not shown), using the structure of the calcium-free human Sorcin; then we used SCBD monomer to solve CaSor. Ca-SCBD crystallized in orthorhombic space group and the Matthews coefficient calculation indicated a dimeric asymmetric unit. The first attempts to solve the phase problem for Ca-SCBD using the whole apo-Sorcin dimer were unsuccessful, suggesting a wide conformational variation. Based on previous published results we expected that the variation regarded mainly the EF1–2–3 subdomain. For this reason we performed the rotational and translational searches with a truncated apo-dimer including E-F-G-H helices (EF4–5 plus part of EF3), finding a partial solution. We fixed this solution and repeated the search using the rest of the apo-model (helices A, B, C, D). Refinements were performed using the maximum-likelihood method with the program REFMAC^47^ and model building with the program Coot^48^. The quality of the models was assessed using the program PROCHECK^49^. The structure of apoSor was refined to 2.1 Å resolution. The final model contains 168 residues (residues 30–198), 73 water molecules, 5 sulfate ions. The structure of CaSor was refined to 1.65 Å resolution. The final model contains 172 residues (residues 26–198), a six residues long peptide, 124 water molecules, 1 sulfate ion, 3 Ca(II) ions with full occupancy and 3 PEG molecules for each monomer.

### PDB accession codes

The coordinates for apoSor have been deposited in the Research Collaboratory for Structural Bioinformatics (RCSB) PDB with accession code 4UPG. The coordinates for CaSor have been deposited in the RCSB PDB with accession code 4USL.

### Phage Display

We used a phage library displaying 16mer randomized peptides (diversity 4 × 10^10^) on the p8 protein flanked by spacer linkers at the N- and C- termini (SSSG- and GGGSGG, respectively). The library is similar to the previously established C-terminal library^50^, but displaying internal, instead of C-terminal, peptide stretches. Phage selections were performed in 5 rounds following the detailed protocols in ref. 51. To assess a potential calcium dependence of the interactions selections were performed in parallel using either 1 mM EDTA or 1 mM CaCl_2_ during the incubation of the phage library with the bait protein as well as in all washing steps. Such an approach has previously been successfully used for the identification of calcium-dependent interactions for the calcium-binding protein S100B^52^. Clonal analysis and sequencing was performed as previously described^53^.

### Cell cultures

Mouse 3T3-L1 preadipocyes (ATCC^R^ CL-173^TM^, American Type Culture Collection) and 3T3-L1 adipocytes were grown on plastic dishes or 10-mm glass coverslips using Dulbecco’s modified Eagle’s medium supplemented with 10% calf serum, 4 mM glutamine, 50 mg/l streptomycin, 100 IU/l penicillin and non-essential amino acids at 37 °C in a humidified CO_2_ incubator. 3T3-L1 preadipocytes were differentiated into adipocytes as described by Tafuri, adding 7.5 μM troglitazone in the medium on days 3 and 4 of differentiation^54^.

The mouse α-Sorcin (33–800) was from Zymed, the rabbit α-Sorcin was homemade, the rabbit α-annexin 11 (NB100–78588) was from Novus Biologicals, the rabbit α-annexin 7 (ABIN65268) and the mouse α-PDCD6 (H00010016-M01) were from Abnova.

For immunofluorescence staining, cells were plated and grown on 10 mm glass coverslips, fixed with 2% paraformaldehyde for 20 min, permeabilized with 0.2% Triton X-100 for 10 min and incubated in 50 mM glycine for 30 min more. Primary antibody dissolved in 1% bovine serum albumin was added and allowed to incubate overnight at 4 °C. Primary antibody was removed, wells washed and secondary AlexaFluor 488, 594 or 647 was added and incubated for 1 h at room temperature. Conventional immunofluorescence and confocal microscopy were performed using confocal LSM710 vertical and Axiovert135M microscope (Zeiss).

### Surface Plasmon Resonance experiments

Surface Plasmon Resonance (SPR) experiments were carried out using a SensiQ Pioneer system. The sensor chip (COOH5) was activated chemically by a 35 μl injection of a 1:1 mixture of N-ethyl-N′-(3-(diethylaminopropyl)carbodiimide (200 mM) and N-hydroxysuccinimide (50 mM) at a flow rate of 5 μl/min. Ligands, i.e. PDCD6 and the N-terminal domain of PDCD6 (KMAAYSYRPGPGAGPGPAAGAALP; a lysine residue has been added to the sequence to ensure peptide immobilization principally via the N-terminus), were immobilized on activated sensor chips via amine coupling. The immobilizations were carried out in 20 mM sodium acetate at pH 4.5; the remaining ester groups were blocked by injecting 1 M ethanolamine hydrochloride (35 μl). Proteins interacting with the ligands (in 10 mM Hepes pH 7.4, 150 mM NaCl + 0.005% surfactant P20) were injected on the sensor chip at a constant flow (30 μl/min). The same procedure was set using the buffer with CaCl_2_ at 100 μM concentration, or with 1 mM EDTA. The increase in RU relative to baseline indicates complex formation; the plateau region represents the steady-state phase of the interaction, whereas the decrease in RU represents dissociation of Sorcin or SCBD from immobilized ligands after injection of buffer. Regeneration procedures are based on two long (2000 s and 500 s) injections of buffer, separated by a brief (5 s) injection of 10 mM NaOH. The sensorgrams were analysed using the SensiQ Qdat program.

## Additional Information

**How to cite this article**: Ilari, A. *et al.* Structural basis of Sorcin-mediated calcium-dependent signal transduction. *Sci. Rep.* **5**, 16828; doi: 10.1038/srep16828 (2015).

## Supplementary Material

Supplementary Information

## Figures and Tables

**Table 1:** Crystal parameters, data collection statistics and refinement statistics of Sorcin in the apo form (apoSor) and in complex with Ca^2+^ (CaSor).

PDB code	apoSor	CaSor
	4UPG	4USL
Space group	I422	C222_1_
Cell parameters (Å)	a = b = 106.4, c = 77.5 Å	a = 52.4, b = 111.6, c = 60.5
Asymmetric unit (residues)	Monomer (30–198)	Monomer(12–17, 26–198)
N° of bound ions	—	3 Ca^2+^
Resolution ranges (Å)	2.1–50.0 (2.1–2.2)	1.65–50 (1.65–1.69)
Unique reflections	23604 (4375)	41040 (3051)
Completness (%)	99.7 (98.2)	99.5 (99.6)
Redundancy	6.8 (7)	3.4 (3.3)
R_merge_ (%)	11 (59)	4 (66)
CC(1/2)	99.8 (88.2)	99.9 (83.3)
I/σ(I)	14.8 (3.6)	21.57 (3.0)
Resolution ranges (Å)	2.10–40.59 (2.10–2.15)	1.65–37.34 (1.65–1.69)
R_cryst_(%)	18.1 (23.4)	19.1 (32.9)
R_free_(%)	21.9 (30.0)	22.1 (33.3)
rms (angles) (°)	1.46	1.32
Rms (bonds) (Å)	0.01	0.01
Wilson B-factor (Å^2^)	29.3	21.6
Residues in core regions of the Ramachandran plot (%)	98.8	99.4
Residues in allowed regions of the Ramachandran plot (%)	1.2	0.6

Values in parentheses are for the highest-resolution shell.

**Table 2:** Effect of ion binding on EF-hands in PEF proteins.

*Prot (pdb)*	*EF1 (θ) [A-B]*	*EF2 (θ) [C-D]*	*EF3 (θ) [D-E]*	*EF4 (θ) [F-G]*	*EF5 (θ) [G-H]*	D-helix vs G-helix *(θ)*
	***θ***	***θ***	***θ***	***θ***	***θ***	
hSorcin						
**ApoSor** (4UPG)	40.8°	60.7°	52°	47.3°	32.9°	57.5°
**CaSor** (4USL)	59.2°	57.9°	66.5°	45.9°	29.9°	78.1°
	***+Ca***	***+Ca***	***+Ca***	—	—	
Δ (+Ca)	**+18,4°**	−2.9°	**+14.5°**	−1.40°	−3°	**+20.6°**
hPDCD6						
PDCD6-**apo** (2ZND)	68.8°	51.5°	59.2°	56.5°	35.8°	80.2°
PDCD6+**Ca** (2ZN9) A = B	72°	52.6°	64°	58.4°	27.2°	82.2°
	***+Ca***	—	***+Ca***	—	***+Ca***	
	+3.2°	+1.1°	+4.8°	+2.1°	**−8.6°**	**+2°**
hPDCD6-**Zn-Alix**(2ZNE)	67.2	55.4	62.4	60.5	A = 31.7 B = 36.6	A = 74.5 B = 75.4
	**+Zn**	—	**+Zn x2**	—	**+Zn**	
Δ(+Zn, +pep)	−1.5°	+4°	+3°	+4°	A = −4°	A = −5.7°
					B = +1°	B = −4.8°
rCalpain-dVI						
**apo** mon**A** (1AJ5)	40.3°	53.6°	30°	51.5°	28°	47°
**apo** mon**B** (1AJ5)	41.2°	52° (54°)*	33.6° (28.5°)*	44.5°	20.5°	48.6°
+ **Ca** (1DVI)	61°	63° (71°)*	46.2° (42°)*	49.4°	23.4°	52.3°
	**+Ca**	**+Ca**	**+Ca**	—	—	
Δ (+Ca)	**+20.7°**	+9°	**+16°**	+1.6°	−3°	**+5.3°**
	**+19.7°**	+11°	**+12°**	+3°	+4°	**+3.7°**

The angle between the helices has been calculated with PyMol. The angle between helices D and G is reported (*θ*), as an indicator of the movement of sub-domain EF1-EF2-EF3 with respect to EF4-EF5.
