# Supplementary material for: Structural basis of Sorcin-mediated calcium-dependent signal transduction
Source: Sci Rep. 2015 Nov 18;5:16828. doi: 10.1038/srep16828 (PMC4649501; doi:10.1038/srep16828)
Supplement: Supplementary Information [file srep16828-s1.pdf]

Structural basis of Sorcin-mediated calcium-dependent signal transduction *

Andrea Ilari[1][#], Annarita Fiorillo[1]*, Elena Poser[1], Vasiliki S. Lalioti[2], Gustav N. Sundell[3], Ylva Ivarsson[3], Ilaria Genovese[1], and Gianni Colotti[1#]*

[1] Institute of Molecular Biology and Pathology CNR; Dept. Biochemical Sciences, Sapienza University, P.le A. Moro 5, 00185, Rome, Italy.

[2] Centro de Biología Molecular Severo Ochoa, CSIC -Universidad Autónoma de Madrid, Departamento Biología Celular e Inmunología, Cantoblanco; Centro de Investigación Biomédica en Red de Enfermedades Hepáticas y Digestivas (CIBERehd), Madrid, Spain.

[3] Department of Chemistry-BMC, Uppsala University, P.O. Box 576, 751 23 Uppsala, Sweden.

*Running title: *Structural Basis of Sorcin Calcium-dependent Activation*

To whom correspondence should be addressed: Andrea Ilari: email: andrea.ilari@uniroma1.it; Gianni Colotti: email: gianni.colotti@uniroma1.it. Institute of Molecular Biology and Pathology CNR; Dept. Biochemical Sciences, Sapienza University, P.le A. Moro 5, 00185, Rome, Italy, Tel: +39-06-49910910; Fax: +39-06-4440062.

Supplemental Figure S1: Residues exposure variation upon calcium binding. **(A)** GetArea and Areaimol give the value of percentage of SAS (%SAS) per-residue, calculated as the ratio between SAS area of a residue X in its three-dimensional structure and SAS of its extended tripeptide Gly-X-Gly conformation. For each residue the difference of %SAS between CaSor and apoSor has been calculated and plotted to identify the surface regions with the main variations. Positive values indicate that the residue is more exposed in CaSor than in apoSor and *vice versa*. **(B)** Only the hydrophobic component of the surface is taken into account. Some artifact has arisen from the absence of side chains in the model (Arg175, Gln107).

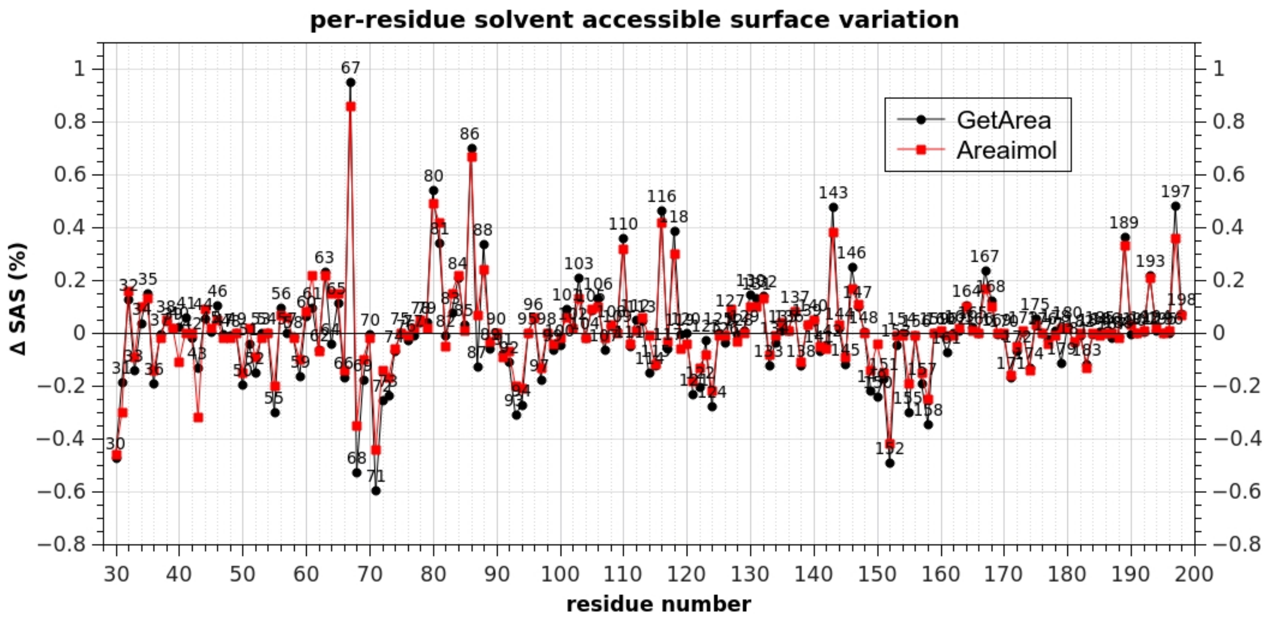

A

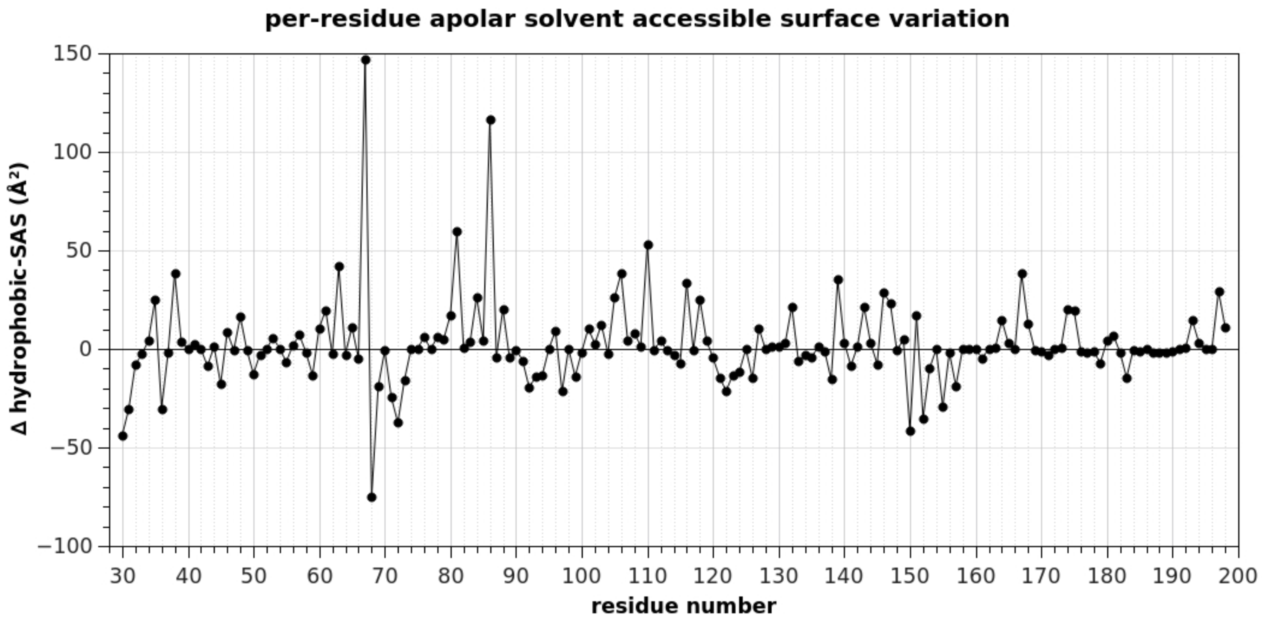

B

Supplemental Figure S2: Two views of the omit map (Fo-Fc, contoured at 3σ) of the peptide, shown as blue lines. The residues Trp105 and Glu97 are shown as reference.

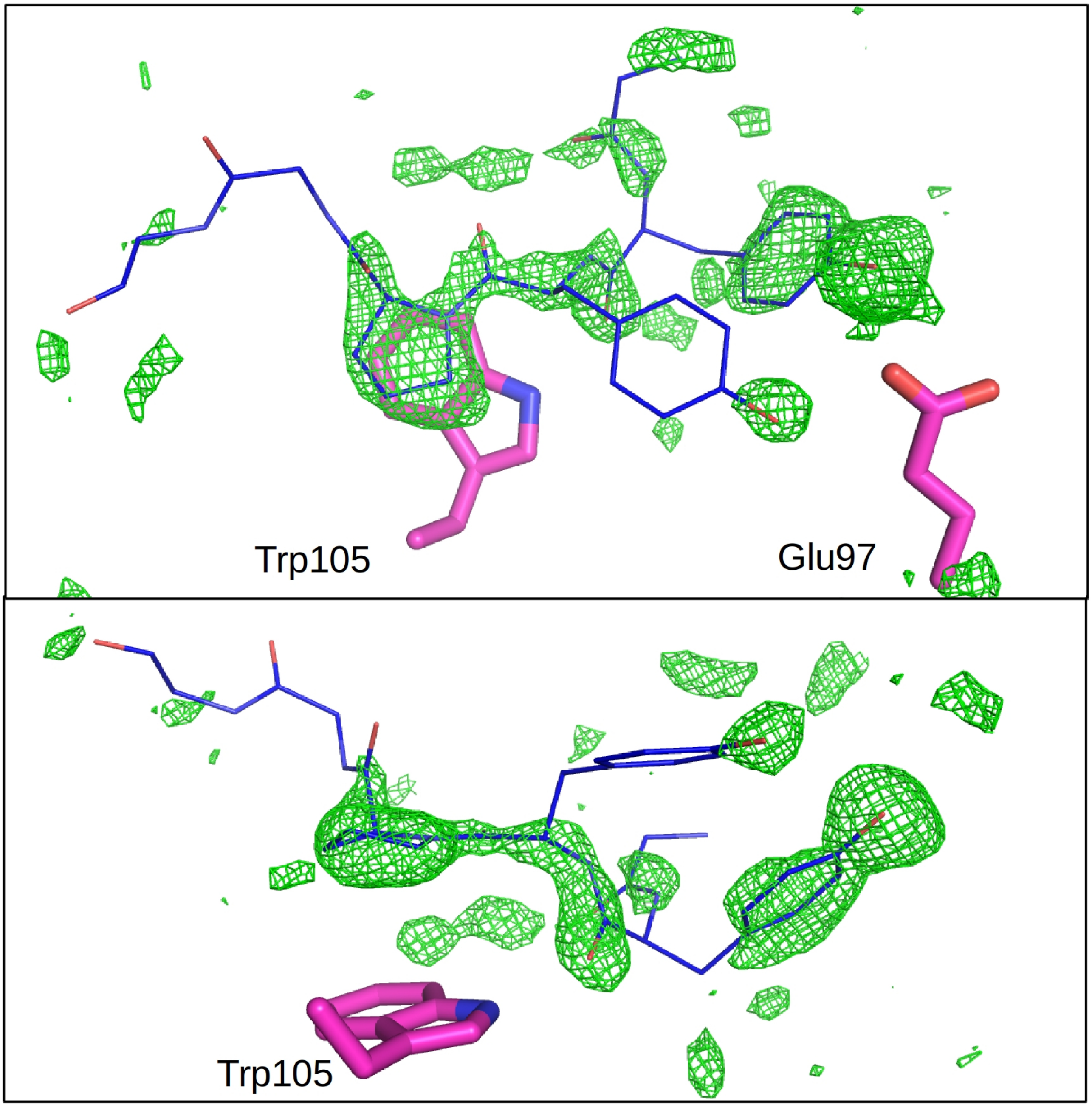

Figure S3: Comparison between the dimeric structures, both in free and calcium bound form, of Sorcin, PDCD6 (pdb: 2znd, 2zn9) and Calpain-dVI-domain (pdb: 1aj5, 1dvi). The structures are presented as transparent surface and cartoon. Three views are given and the odd EF-hands are colored (EF1: cyan; EF3: green; EF5: magenta).

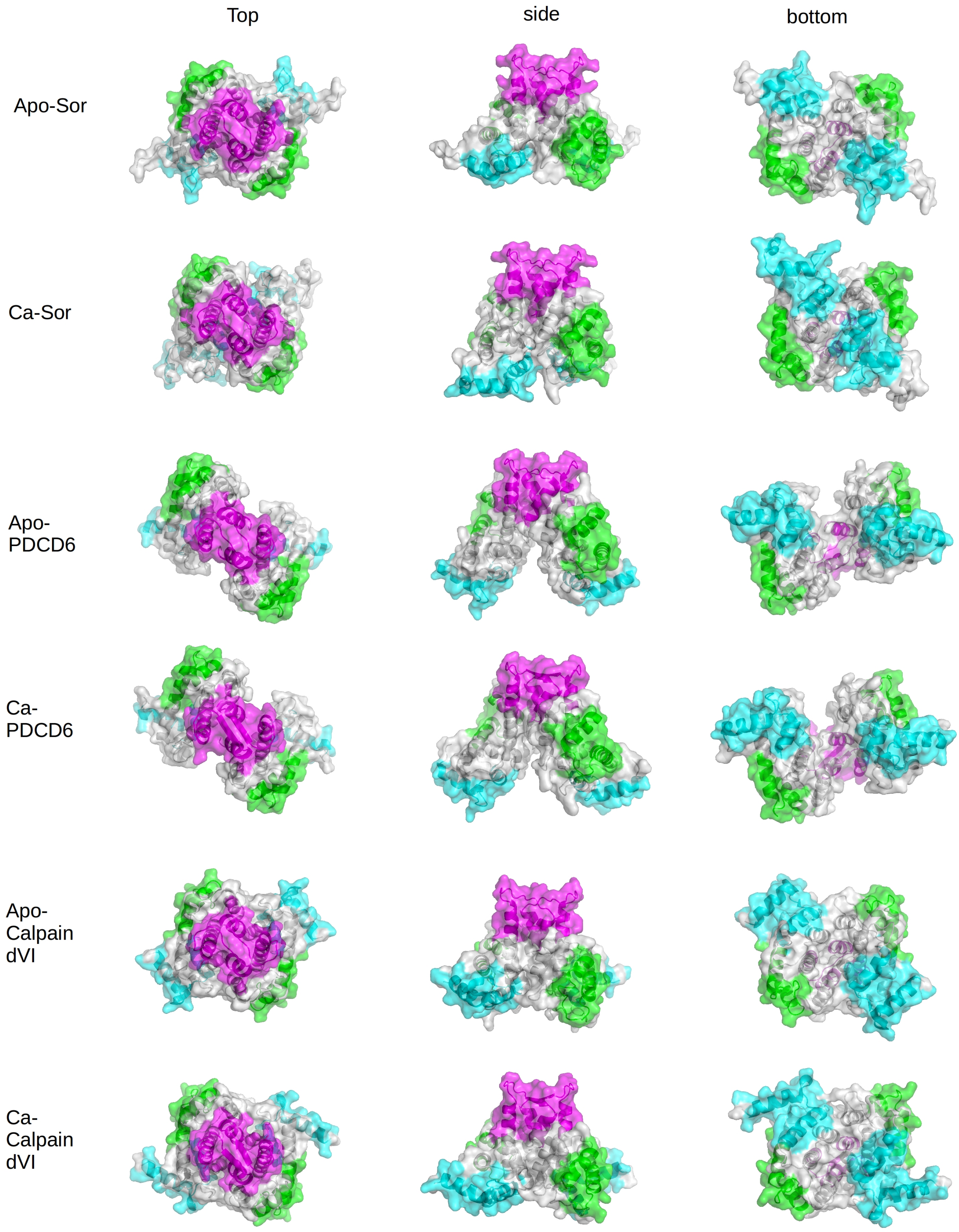

Supplemental Figure S4. Peptides showing interaction with Sorcin, selected in phage display experiments, obtained in presence of Ca^{2+} and/or EDTA. # indicates the number of sequenced clones of a given sequence. Consensus is shown in Figure 7.

Peptide	# 1 mM Ca^{2+}	# 1 mM EDTA
Group 1:		
DTSWYWWVPWGDGYST	1	0
PAYSMWFPWMPAISYV	1	0
GWWWFPQNHILGNMVS	1	0
QSYLWWWQPPVSIISA	1	0
SWWWEPMDNPWTFYVT	1	0
WWWQWWPFQEPMSVMM	1	0
SVAWWYPWERWQSITM	1	0
AYSRWWFPYMYEVVGS	1	0
VEEMWTWPYIQYMIQT	1	0
PEWHPLTREMMWEILR	1	0
MWWWWAPSEVIPRHLS	1	0
GNAGFFWHWWGPRLES	1	0
VYMMPWSDFMQQWQQI	1	0
YDPSWMDQHFGWQSLV	1	0
GFSEHWFSPWTAWVTE	1	0
GSWWGLATFQQFDPFV	1	0
PVWLAPEAHEVMQYYF	1	0
VWTGYDPMNITTWIFM	1	0
HQWEYWHWPWQLASGM	1	0
TWMGTPWWWVGGVMQI	1	0
WPFWWWIPGPQYAAAS	0	3
PSEWWWWRPLGMTEMV	0	2
DTSWYWWVPWGDGYST	0	1
SIGAWWWGPMQDYVHV	0	1
WPVYWWGPLAMQLDYV	0	1
FMLWNWATWPWITPHQ	0	1
WSREWWQAIFSWPTWY	0	1
AYWWPVNSWFVEYVPT	0	1
MWQWWGVPHNMWLDEI	0	1
YTGFMSMWPELWQPVG	0	1
FYPWMWMHQGWDLVLP	0	1
MYWMDPLYGWWWEMYQ	0	1
YWWHEFQPWPNMDILE	0	1
WYGIPFWNFWIQTSWS	0	1
Group 2:		
QHDMYSQWYTLVSTMV	2	1
PHELWLTHFPWMDMIQ	2	0
VVSTDNYMSWHDFMMW	1	0
MPEVITWETFYEYMGH	1	0
TGDAPMDWLYQWQNVM	1	0
FAQEGPEDWFDFWTVV	1	0
EMWADWYILMAEMENV	1	0
WTERDMFLMNLWSMQM	1	0
WGQDDFMMMNEWIKHA	1	0
WTLEDWLAMNQMASMT	1	0
VGDMDTWDWNWLLTWE	1	0
ELDWFNYMVSDQWRMV	1	0
YNDAEYWQNLWEFTKY	1	0
MPPLISSDWMWTVMMT	1	0
IEQWYEMMMSLQADHI	1	0
AWDWWGTDNDWPDWFV	1	0
VDHWDVWQEMARVMYQ	0	1
WMNFVDWLYWRKHTIE	0	1

Table S1. Ion coordination in CaSor. x and -x indicate the axial ligands, y, -y, z, -z are the equatorial ones, -z is the bidentate ligand in CaSor. The atoms that directly bind ions are in brackets, in the case of the -x ligand the residue forming a hydrogen bond with H_2O is indicated. The distance between Ca^{2+} and each ligand is reported.

		x	y	z	-x	-y	-z
CaSor	EF1	Ala43 (O)	H_2O	Asp46 (OD1)	H_2O (Asp50)	Gln48 (O)	Glu53 (OD1-OD2)
		2.35	2.45	2.36	2.42	2.30	2.49, 2.59
	EF2	Asp83 (OD1)	Asp85 (OD1)	Ser87 (OG)	H_2O (Gln48)	Thr89 (O)	Glu94 (OD1-OD2)
		2.34	2.36	2.57	2.36	2.43	2.51, 2.54
	EF3	Asp113 (OD1)	Asp115 (OD1)	Ser117 (OG)	H_2O (Asp121)	Thr119 (O)	Glu124 (OD1-OD2)
		2.33	2.39	2.34	2.26	2.28	2.62, 2.53

Table S2. Rmsd of known structures of Sorcin with respect to apoSor. The structures have been superimposed, both using the Cα atoms of residues 112-198 (LSQ method) and the Cα atoms of all the protein residues (SSM method) by the program COOT, apoSor has been used as reference structure.

	Whole structure (SSM)	112-198 (LSQ)
CaSor	3.13	1.52
F112L	---	0.73
1JUO, A	1.05	0.73
1JUO, B	2.38	1.30

Table S3. Solvent accessible surface (SAS) area for CaSor and apoSor. Two different programs, i.e. Areaimol and GetArea, with different algorithms have been used with default settings. GetArea allows to discriminating between hydrophobic and hydrophilic contributions. The calculations have been performed on the dimers, taking into account the residue range 30-198 and excluding ligands other than calcium.
Although the programs give slightly different results, the solvent accessible surface increases of about 5% upon calcium binding, with a major hydrophobic component.

	Areaimol results	GetArea results		
	total (Å^2)	apolar (Å^2)	polar (Å^2)	total (Å^2)
ApoSor	15762	8930	6552	15482
CaSor	16610	9757	6462	16218
Variation upon Ca^{2+} binding	*848*	*827*	*-90*	*736*
